# Effect of urbanization and its environmental stressors on the intraspecific variation of flight functional traits in two bumblebee species

**DOI:** 10.1007/s00442-022-05184-x

**Published:** 2022-05-16

**Authors:** Nicola Tommasi, Emiliano Pioltelli, Paolo Biella, Massimo Labra, Maurizio Casiraghi, Andrea Galimberti

**Affiliations:** 1 ZooplantLab, Department of Biotechnology and Biosciences, University of Milano-Bicocca, Milan, Italy; 2 INFN Sezione Di Milano Bicocca, Milan, Italy

**Keywords:** Functional diversity, Urban heat island, Pollination, Urban ecosystems, Wing asymmetry, Geometric morphometrics

## Abstract

The way urbanization shapes the intraspecific variation of pollinator functional traits is little understood. However, this topic is relevant for investigating ecosystem services and pollinator health. Here, we studied how urbanization affects the functional traits of workers in two bumblebee species (*Bombus terrestris* and *B. pascuorum*) sampled in 37 sites along a gradient of urbanization in North Italy (an area of 1800 km^2^ including the metropolitan context of Milan and other surrounding capital districts). Namely, we investigated the effect of land use composition, configuration, air temperature, flower resource abundance, and air pollutants on the variation of traits related to flight performance and of stress during insect development (i.e., wing size, wing shape and size fluctuating asymmetry). The functional traits of the two bumblebees responded idiosyncratically to urbanization. Urban temperatures were associated with smaller wing sizes in *B. pascuorum* and with more accentuated fluctuating asymmetry of wing size in *B. terrestris*. Moreover, flower abundance correlated with bigger wings in *B. terrestris* and with less asymmetric wing size in *B. pascuorum*. Other traits did not vary significantly, and other urban variables played minor effects. These species-specific variation patterns highlight that environmental stressor linked to urbanization negatively impact the traits related to flight performance and development stability of these syntopic bumblebees, with possible consequences on the pollination service they provide.

**Supplementary Information:**

The online version contains supplementary material available at 10.1007/s00442-022-05184-x.

## Introduction

Widespread phenomena of urbanization are driving deep changes on landscape features, their temperature and pollutants, creating conditions that impact biodiversity (Foley et al. 2005; Weng et al. 2007; Wenzel et al. 2020). Plants and animals can respond to these environmental variations by shifting their distribution (Colla et al. 2012), phenology (Huchler et al. 2020), and/or shaping some morphological traits considered “functional”, *i.e.* relevant for their ecology, fitness and behavior (Alberti et al. 2017; Eggenberger et al. 2019; Nooten and Rehan 2020). In bees, trait variation due to environmental alteration could affect the efficiency of the pollination ecosystem service they provide though impacting the way they interact with plants (Buchholz and Egerer 2020; Biella et al. 2019a, b). Environmental alteration could also impact bumblebees development, for example, by limiting the abundance of floral resources due to the increasing proportion of anthropized surfaces (Steffan-Dewenter et al. 2001). This scenario, in turn, could trigger body size decline due to less food supplied to larvae (Couvillon et al. 2010), with consequences for bumblebee task allocation, provision loads and ultimately fitness (Goulson et al. 2002; Foster et al. 2004). Furthermore, landscape anthropization could change the local climate, thus altering pollinator ecology, development and foraging (Radmacher and Strohm 2010). Specifically, the higher degree of cemented “impervious” land cover that characterizes urban areas is often associated with increasing temperatures, a phenomenon known as the “heat island effect” (Chun and Guldmann 2018). Observations from previous studies have strengthened the hypothesis that pollinator insects could face a shift toward smaller body size as an adaptation to reduce the risk of overheating while foraging in warmer conditions (Peters et al. 2016; Gérard et al. 2018a). Considering the worldwide steady growth of cities size (Sun et al. 2020), new insights on pollinator responses are necessary.

Previous studies investigated the morphological responses of pollinators to anthropogenic pressures, mainly focusing on body size (e.g., Chown and Gaston 2010; Eggenberger et al. 2019; Theodorou et al. 2021). In bees, this character responds rapidly to environmental changes (Chown and Gaston 2010), it shows little heritability, and its variation mainly depends on the amount of food received during the larval development (Couvillon et al. 2010). Bee size is positively correlated with the foraging range (Greenleaf et al. 2007). Generally, larger bees show more efficient flight performances (Harrison and Roberts 2000), since flight muscle ratio is known to increase with body size in flying insects (Samejima and Tsubaki 2010). Size also determines the metabolic rate and resource needs of adult imagos, with larger bees having higher metabolic rate (Kelemen et al. 2019) and thus potentially being more susceptible to shortage in floral resource availability (Couvillon and Dornhaus 2010). However, to date, the investigation of pollinators body size variation in anthropogenic habitats yielded heterogeneous results. A recent study on bumblebees found bigger workers in cities (Theodorou et al. 2021). This study speculated that such a pattern is an adaptation to longer flights for collecting resources, particularly in view of the severe green patches fragmentation of urban landscapes (Greenleaf et al. 2007). Conversely, a study by Eggenberger et al. (2019) found smaller bumblebee foragers in cities. This was interpreted as an effect of both limited local resource abundance and warmer temperature in urban areas. Given these contradicting results and different interpretations, more studies are needed for clarifying the existing patterns of pollinator morphological responses to urbanization.

Beyond body size, wing size and shape are important functional traits in bumblebees and more in general in pollinator insects. This is because wing size is believed to be related to flight length and it influences metabolic costs (Fernandez et al. 2017; Soule et al. 2020), while shape is considered important for flight maneuverability (Kolliker et al. 2003; Grilli et al. 2017). Indeed, morphometric analysis are gaining in importance for quantifying even subtle variations in such morphological traits. These variations are usually informative of stress exposure, and thus provide information about animal population health status (Adams et al. 2001). One of the advantages of using trait variation to measure stress is that changes of phenotypes are detectable before an overall decrease in population viability (Hoffmann et al. 2005). Therefore, quantifying traits variation could become an essential practice when evaluating local and landscape-level stressors. A metric that has grown in popularity is the fluctuating asymmetry (FA) (Klingenberg 2001; Beasley et al. 2013; Alves-Silva et al. 2018), defined as the presence of small, randomly placed deviations from perfect bilateral symmetry due to the occurrence of developmental instability, driven by exogenous environmental conditions (Klingenberg 2015). FA differs from another type of bilateral asymmetry, the directional asymmetry (DA), that occurs when the two sides are steadily different with a predictable direction to this difference. While DA has a genetic basis and therefore could be less impacted by the environment (Palmer and Strobeck 2003), the FA is considered a valid proxy of stress exposure to conditions that typically occur in urban environments (e.g., higher temperature and air pollutants) (Beasley et al. 2013). For instance, laboratory-based studies have demonstrated that higher CO_2_ level or low temperature led to an increase in wing FA, supporting the possible role of traffic pollutants and climatic variation in determining developmental instability (Klingenberg et al. 2001; Hoffmann et al. 2002). However, asymmetries could be found in wing shape and/or in wing size and they even show different responses to the same stressor. For instance, in a recent study by Gérard et al. (2018b), variations in wing size asymmetry were observed in response to thermic and parasitic stress while these same stressors caused no alteration in wing shape asymmetry level. To characterize the effects of urbanization and of the related environmental stressors on pollinator insects, we quantified the morphological variation in two species of bumblebee (*i.e.*, *Bombus pascuorum* and *B. terrestris*). The two species were selected as they are among the most common and widespread pollinators in Europe (Pekkarinen and Teräs, 1993; Rasmont et al. 2008) and have been largely used as model species in many studies related to the effects of urbanization or other stressors (Eggenberger et al. 2019; Theodorou et al. 2021). We sampled foraging workers from populations spanned across a gradient of growing urbanization (from seminatural areas to highly urbanized sites) in Northern Italy, a region that experienced a strong anthropogenic footprint (Perini and Magliocco 2014; Salata 2017). We expected to find quantitative variation in bumblebee functional traits of body size and wing FA in response to several facets of urbanization. First, we tested associations with increased fragmentation of green patches that is often found in urban landscapes (Li et al. 2019). We also tested responses due to environmental stressors amplified by urbanization, such as increased temperatures (Feng et al. 2014) and pollutants (Salahodjaev 2014), and decreased floral resource abundance (Ushimaru 2014). Regarding body size, we based our survey on two alternative expectations that emerged from previous studies. On one hand, one could expect to observe an increase in body size if green patches fragmentation triggered an adaptation to increase foraging ranges, as suggested by (Warzecha et al. 2016). Alternatively, a reduction in body size could arise as a way to reduce the risk of overheating in warmer urban habitats (Maebe et al. 2021; Pereboom and Biesmeijer 2003) or as a consequence of limited floral resources (Chown and Gaston 2010). Regarding wing FA in shape and size, we expected to find increased FA in response to higher levels of biotic and abiotic stressors that are expected to occur in more urbanized landscapes, such as limited floral resources, temperature, and air pollutants.

## Materials and methods

### Study species

This study was focused on two co-occurring species of bumblebee: *Bombus terrestris* (Linnaeus 1758) and *B. pascuorum* (Scopoli 1763). Both species are pollinators common in Europe and can be easily found while foraging in different habitats (Polce et al. 2018), even in urban areas (Meeus et al. 2021; Banaszak-Cibicka, and Żmihorski 2012), including the surveyed region (Intoppa et al. 1995). Given these characteristics, these species are reliable to investigate responses by pollinating insects to landscape anthropization (Eggenberger et al. 2019; Theodorou et al. 2021). Using two different, albeit related, species could even clarify if the observed patterns are general or rather shaped by different life histories. The two selected species, in fact, have slightly different foraging ranges, with an estimated maximum of 449 and 758 m for *B. pascuorum* and *B. terrestris*, respectively (Knight et al. 2005). Nesting habits are also dissimilar as *B. terrestris* builds its nest in subterranean holes, while *B. pascuorum* on top of or slightly beneath the soil surface (Goulson 2010). Another important difference is represented by their dietary regimes, since *B. pascuorum* usually have a narrower trophic niche and a preference for deep-corolla flowers (Harder 1985) while *B. terrestris* is highly polylectic (Dafni et al. 2010; Biella et al. 2019a, b).

### Study design and sampling

**Fig. 1:**
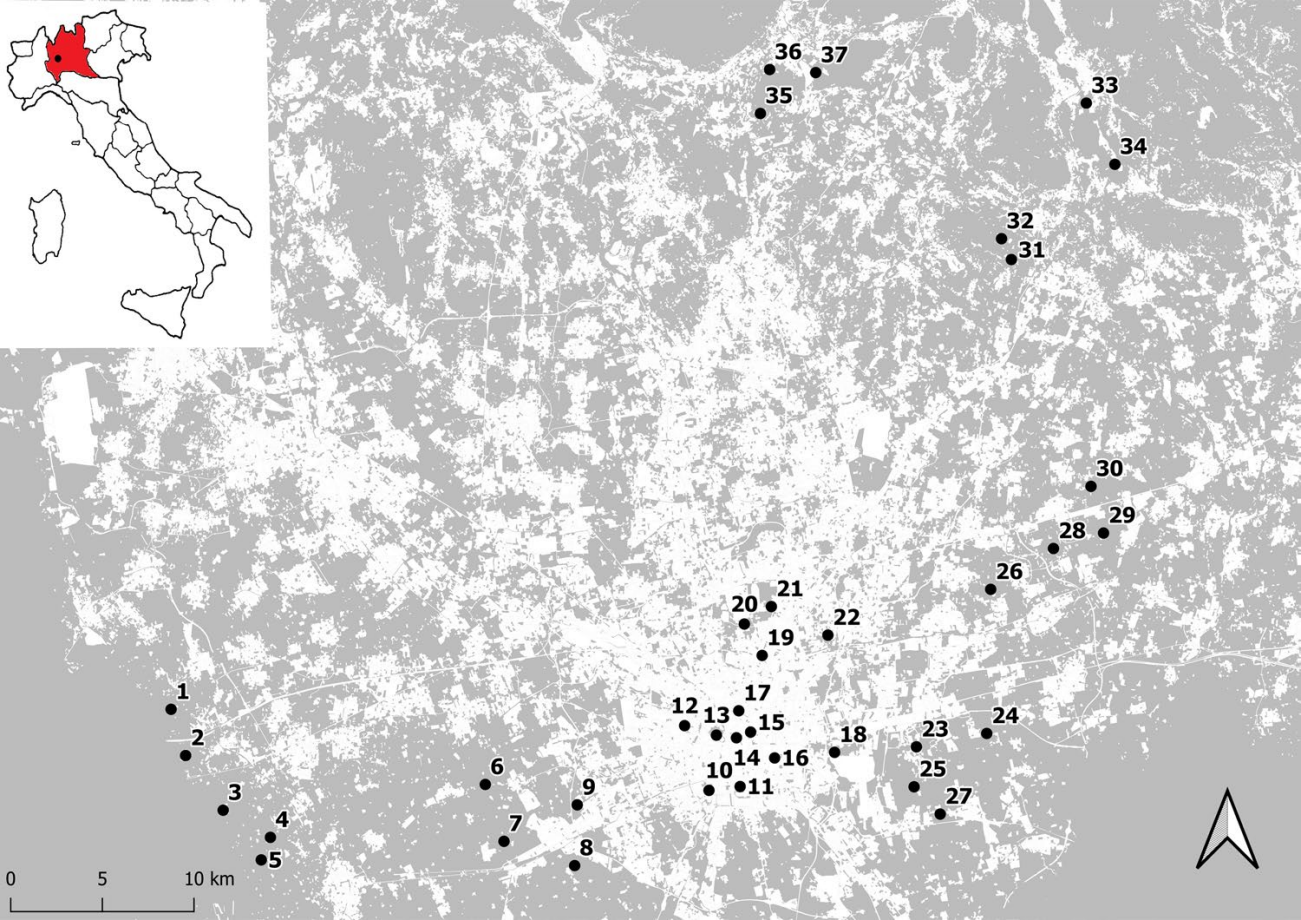
Map showing the distribution of the sampling sites along the urbanization gradient. White areas correspond to cemented surfaces

Samplings were conducted at 37 sites (Fig. 1), in July 2019, between 9:00 and 12:00 only on days with sunny and windless weather conditions. The study sites were distributed within an area of about 1800 km^2^ covering four administrative provinces of northern Italy (i.e., Milano, Monza e della Brianza, Lecco and Como). A minimum distance between sites of 1 km was imposed to avoid the non-independence of sites (Phillips et al. 2019) since it is above the maximum foraging range observed for the two species (Knight et al. 2005). The vast majority of the sites were farther than 2 km each other and were selected along a gradient of growing urbanization, ranging from areas highly dominated by seminatural hay meadows close to forest with little urban areas nearby, to sites characterized by a high degree of impervious surface (i.e., concrete, building, and asphalt). To select sampling sites, impervious surfaces were mapped in a GIS software based on a regional land use cartography (2018-DUSAF 6.0; https://www.dati.lombardia.it/Territorio/Dusaf-6-0-Uso-del-suolo-2018/7rae-fng6). This land use cover map is available at the scale of 1: 10,000 with a minimum linear dimension of polygons of 20 m and was developed from AGEA orthophotos and SPOT 6/7 satellite images. Sites were chosen on a visible gradient of growing impervious cover. Once identified as suitable, the land use composition in the surrounding of the candidate sampling sites was confirmed using satellite images and/or direct field surveys. For each species, five to six specimens were captured while foraging inside a plot of about 50 m × 50 m at each site using an entomological net. After having excluded queen, males, and specimens presenting damaged wings, 179 *B. pascuorum* (mean per site = 4.8 ± 0.4) and 169 *B. terrestris* (mean per site = 4.5 ± 0.3) were subjected to morphometric analyses. After collection, the insects were stored at − 80 °C until further analyses.

### Landscape and environmental variables description

The previously mentioned land use cartography was adopted to quantify landscape urbanization around sampling sites. Through QGIS 3.10.11, a 1 km radius buffer area was created around each site where landscape composition was evaluated arranging DUSAF original level and sub-level of land use classification into two macro categories: impervious (i.e., buildings, infrastructures, roads, and cemented surface), and seminatural land (i.e., meadows, forests and urban green spaces) (see Online resources, Additional information 2 for a list of DUSAF codes assigned to each grouping). The size of buffer areas was selected according to the previously mentioned maximum foraging range of the two investigated species (Knight et al. 2005).

For each site, the ratio between impervious and green land was computed to quantify the intensity of urbanization within the 1 km buffer area. The gradient of urbanization was also described by green habitat fragmentation, a measure of landscape configuration, that was quantified by computing the edge density (ED), namely the ratio of edge length of green and seminatural patches over their total area (Wang et al. 2014) within the 1 km buffer area of each site. Other environmental biotic and abiotic features, possibly influenced by the urbanization degree, were considered to test for their potential effects on altering body size and wing size/shape FA. Specifically, land surface temperature was calculated as the mean value in the period June-July using data retrieved through remote sensing imaging spectroradiometer (MODIS) MOD11A2 from the NASA database (https://modis.gsfc.nasa.gov/data/dataprod/mod11.php) with a resolution of 1 km. Since the two species of bumblebee studied are characterized by a life-cycle from eggs to adults of about two months (Goulson 2010), the time frame for which these data were taken into consideration should well represent the mean temperature experienced by larvae during their development. The resolution here adopted does not permit to describe microclimatic variation, but it is suitable to infer the broader temperature variation at the landscape scale and the foraging range of the two bumblebee species (Knight et al. 2005). A map reporting the variation of mean temperatures along the investigated landscape is reported in Online resources, Figure S1. Air pollution was estimated as the mean of daily concentrations of NO_2_ over two months (June and July) registered by Regional agency for environmental protection (ARPA). Specifically, data taken from the nearest monitoring stations at each sampling site were used to calculate the mean value of NO_2_ concentrations. (https://www.arpalombardia.it/Pages/Aria/qualita-aria.aspx). A map reporting the location of monitoring stations along the investigated landscape is provided in Online resources, Figure S1.

An expeditive estimation of floral resources at each site (i.e., the total number of flowers) was performed using six quadrats 1 m × 1 m (covering a proportion of sampling area similar to that reported in Fisher et al. 2017) randomly placed in the flowering green spaces within or closest to the sampling area, and counted the number of flowers found there (as in Ushimaru 2014). Flowers were counted considering single or composed inflorescences as units: for *Myosotis* sp., *Galium* sp., and *Capsella bursa-pastoris*, and all Asteraceae the number of inflorescences was counted. The values of the listed landscape and environmental variables in all the sampling sites are reported in Online resources, Table S1 along with histograms showing their variation along the sites (Additional information 1).

### Specimens imaging and wings measurement

**Fig. 2:**
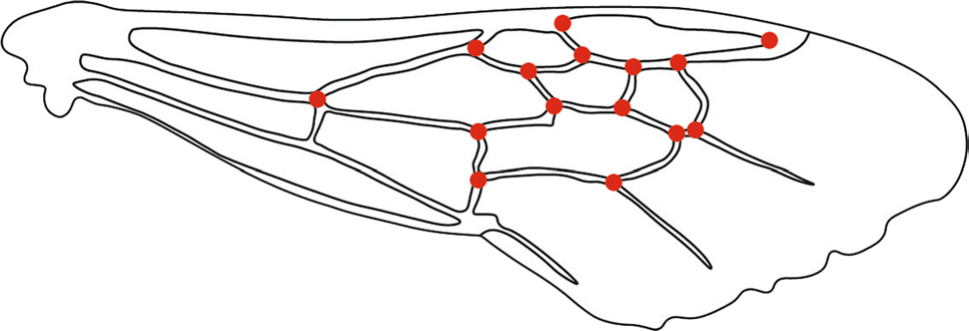
Right forewing of *B. terrestris* with landmark locations used in this study. Details on the formulas applied to calculate centroid size and consequently fluctuating asymmetry from these landmarks are reported in the manuscript section “Specimens imaging and wings measurement” and in the references within

The forewings of all individuals were detached at the base and scanned at high resolution (i.e., 600 dpi). The obtained images were converted into TPS files using tps-UTIL 1.74. This file format follows the standards for geometric morphometrics (Rohlf 2015). TPS file can contain two- or three-dimensional landmark data and the information about the scale factor applied to each specimen is also provided. Once converted into TPS, images were digitized using the tps-Dig 2.31 software (Rohlf 2015), with two-dimensional cartesian coordinates of 15 landmarks positioned at wing vein junction (Fig. 2) (as in Aytekin et al. 2007; Klingenberg et al. 2001). Bumblebees with damaged or badly worn wings were excluded from further analyses.

The analysis of landmark configuration was conducted in MorphoJ 1.07 software (Klingenberg 2011). To remove all the effects of scale, rotation and position, a standard protocol based on a generalized least square Procrustes superimposition was applied (Klingenberg 2011). This strategy permits to obtain a new set of superimposed landmark coordinates (i.e., ‘Procrustes shape coordinates’) describing the wing shape and size features. Wing size was estimated as the centroid size: i.e., the square root of the sum of squared distances from the centroid of each landmark configuration, and used as a proxy of body size (hereafter “body size”, as in Outomuro and Johansson 2011 and Dellicour et al. 2017). To confirm the positive relation between centroid size and body size, the inter tegular distance (IT), another measure of body size used in bees studies (Warzecha et al. 2016), was retrieved from a subset of 50 individuals of each species. Afterwards, the correlation between IT and centroid size was calculated (*B. terrestris* *r* = 0.7, *p* < 0.001; *B. pascuorum* *r* = 0.7, *p* < 0.001). Wing size asymmetry was computed by dividing the absolute difference between left and right centroid sizes by the mean centroid size and multiplying by 100 (Leonard et al. 2018). To estimate wing shape variation, Procrustes distances were computed for each individual (Klingenberg 2015). These represent the measure of an individual’s overall asymmetry (i.e., combining DA and FA components), obtained by taking the square root of the sum of squared distances between corresponding right and left Procrustes’ coordinates (Klingenberg 2015).

### Statistical analysis

According to the protocol outlined in Klingenberg (2015), we first estimated the entity of the measurement error because the levels of asymmetry in bilateral traits are subtle and it could possibly cause considerable variation in the assessment of asymmetry. This was performed by double-scanning the wings and digitizing their landmarks for a subset of 40 specimens, with the Procrustes ANOVA in MorphoJ (Klingenberg 2001; Klingenberg 2015) to evaluate the measurement error. Afterwards, following Costa et al. (2015), to isolate the FA component, we calculated the amount of directional asymmetry (DA) and tested its entity again with the Procrustes ANOVA in MorphoJ considering all the measured specimens in a single analysis. Only if DA occurred significantly, the individual asymmetry measures were corrected by subtracting the mean DA, thus isolating the FA component as in Costa et al. (2015).

To investigate the relationship between morphological traits and covariates, linear mixed models were used. The responses of the two species were assessed separately. In all the models, the ratio between impervious and seminatural surfaces was initially included as a predictor with the other variables. However, variance inflation factor (VIF) criteria was used to assess the absence of collinearity among model variables, and it indicated that the ratio between impervious and seminatural surfaces was highly collinear with the other variables; see also the correlation matrix reported in Online resources, Table S2. Thus, we decided to exclude the ratio between impervious and seminatural surfaces from subsequent models. The other variables, describing landscape configuration, biotic, and abiotic features, as well as the interaction between all these variables, were included in the models following the ecological expectations of our hypothesis. Specifically, changes in body size were evaluated in response to the edge density of green area, temperature, and floral resource availability because they could directly influence body size, with bigger sizes in increasingly fragmented green areas, and/or with more flower resources, and/or less temperatures (Warzecha et al. 2016; Pereboom and Biesmeijer 2003; Chown and Gaston 2010). Concerning wing FA, the temperature, concentration of NO_2_, and flower resources limitation were included in the models following our hypothesis that they could be stressors expected to increase asymmetry (Hoffmann et al. 2002; Klingenberg et al. 2001; Leonard et al. 2018). Sampling site was included as a random effect in all the models. A backward stepwise model selection based on AIC was used to remove variables and their combinations that did not improve the model fit and thus to obtain reliable final models (Zuur et al. 2009). To improve the fit between the predictor and the response variable, log-transformation of the covariate “floral resources” was applied as reported in Table 1. All the analyses were performed using R (version 3.6.1; R CoreTeam 2019).

**Table 1:** Output of Linear mixed models of body size (*N* = 348) and fluctuating size asymmetry (size FA) (*N* = 347) of each species as a function of biotic and abiotic covariates of urbanization, with site identity as random factor. Final models were selected through backward stepwise selection using AIC criterion. ΔAIC reports the difference in AIC values between full and final models. Models and results of shape FA are reported in Online resources, Table S3 as they were non-significant

Species	Response variable	Full model covariates	Final model covariates	ΔAIC	Bi	*χ*2; df	*p* value
*Bombus terrestris*	Body size	Temperature T	log (Floral resources)	16.5	0.025	6.610;1	**0.010**
		Edge density ED					
		log (Floral resources FL)					
		Interaction T × ED × FL					
*B. pascuorum*	Body size	Temperature T	Temperature	17.9	− 0.003	7.403;1	**0.006**
		Edge density ED					
		log (Floral resources FL)					
		Interaction T × ED × FL					
*B. terrestris*	Size FA	Temperature T	Temperature	23.5	0.052	7.183	**0.007**
		log (Floral resources FL)					
		NO_2_ N					
		Interaction T × FL × N					
*B. pascuorum*	Size FA	Temperature T	log (Floral resources)	30.3	− 0.161	6.118;1	**0.013**
		log (Floral resources FL)					
		NO_2_ N					
		Interaction T × FL × N					

Significant *p* values are reported in bold. *βi* regression coefficient, *χ*2 chi square values, *df* degrees of freedom÷

## Results

**Fig. 3:**
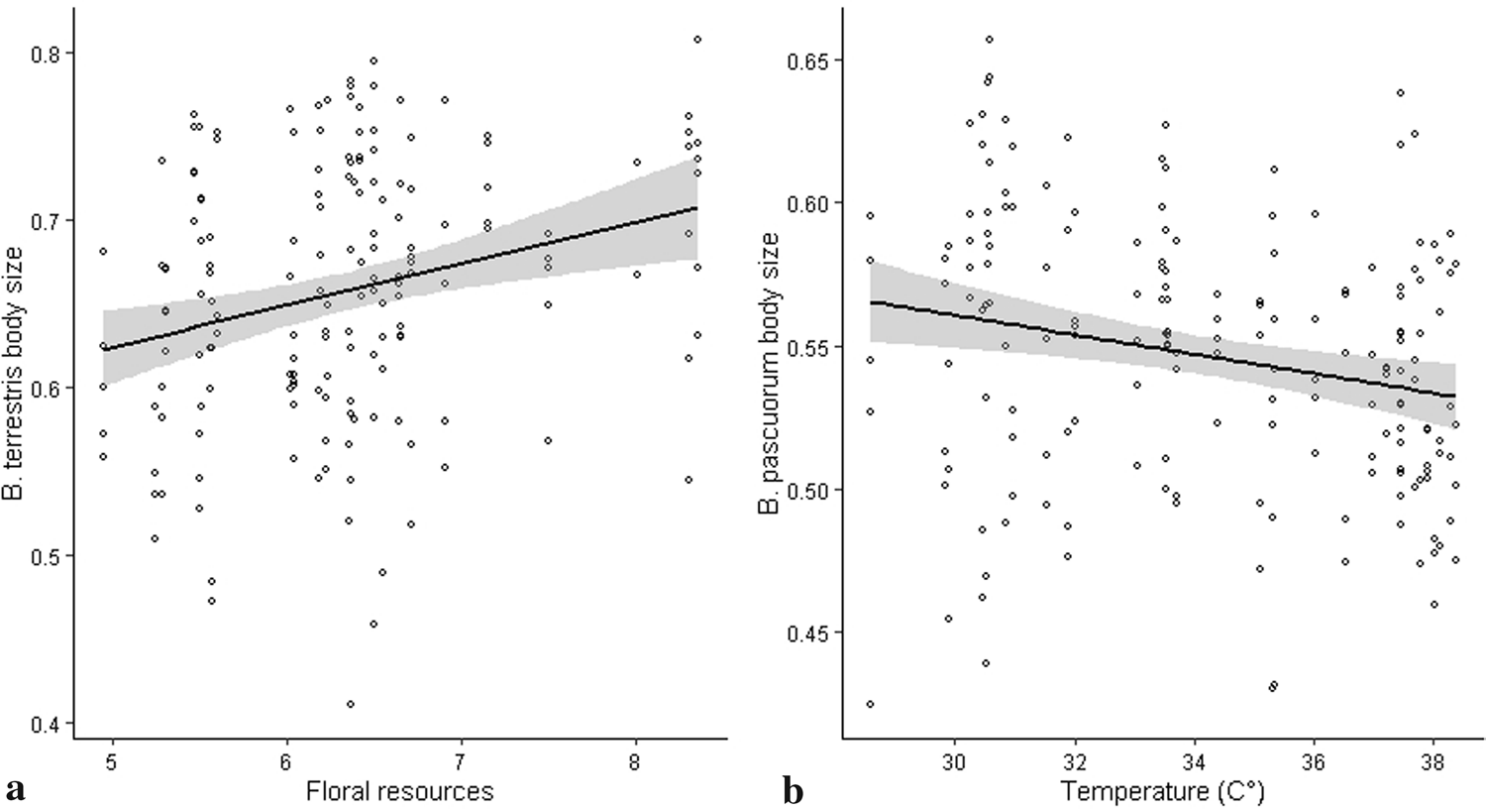
Body size variations (estimated by the centroid size adimensional measure) as a function of **a** Floral resource abundance in *B.terrestris* and **b** Summer temperature in *B. pascuorum*. The black line and grey areas indicate the relationship and its confidence intervals (*α* = 95%) as estimated with Linear mixed models, see methods for further details

The measurement error was negligible because it was not significant for wing size (df = 79, *F = *2.67 *p* = 0.4578, *R*^2^ = 0.0009) and shape (df* = *2054, *F* = 0.51, *p* = 0.9976, *R*^2^ = 0.07), further details in Additional information 3. Different patterns of size variation were found in the two bumblebee species. *B. terrestris* body size was found to increase in response to floral resource abundance by 13.6% along the gradient of this covariate (Fig. 3a, Table1) while *B. pascuorum* body size decreased in response to the increasing temperature by 6.23% along the whole temperature gradient (Fig. 3 b, Table 1).

Concerning wing asymmetry, both species showed a significant level of shape DA (*B. pascuorum* df = 26, *F* = 4.66; *p* < 0.0001, *R*^2^ = 0.008; *B. terrestris* df = 26, *F* = 5.60; *p* < 0.0001, *R*^2^ = 0.009), while size DA was statistically significant only in *B. pascuorum* (df = 1, *F* = 29.77; *p* < 0.0001, *R*^2^ = 0.0007; in *B. terrestris* df = 1, *F* = 0.51; *p* = 0.4779, *R*^2^ = 0.000008). Measures of FA were then obtained by correcting for directional components.

**Fig. 4:**
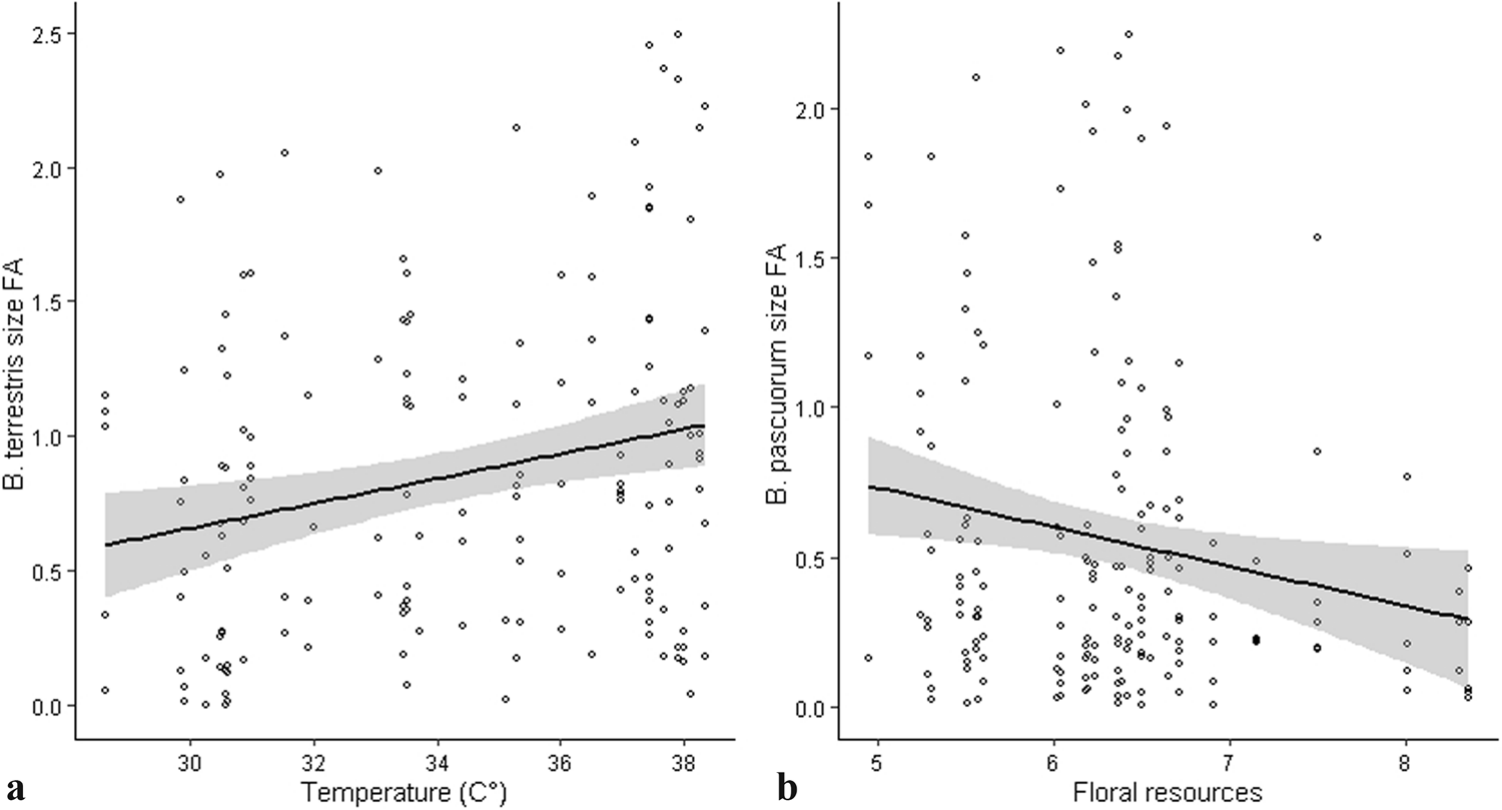
Variations in wing size Fluctuating Asymmetry (FA) as a function of **a** Summer temperature in *B.terrestris* and **b** Floral resource abundance in *B. pascuorum*. The black line and grey areas indicate the relationship and its confidence intervals (*α* = 95%) as estimated with Linear mixed models, see methods for further details

*B. terrestris* size FA was positively correlated to temperature (Fig. 4 a; Table 1) showing an increase of 58.4% from the lowest to the highest temperature values while *B. pascuorum* size FA decreased by 41.3% with higher floral resource availability (Fig. 4 b; Table 1)*.* None of the predictor variables (i.e., temperature, NO_2_, resource abundance, and the interaction among these variables) showed a significant effect on wing shape asymmetry in both bumblebee species (the output of non-significant regression models is available in Online resources, Table S3).

## Discussion

In this study, we quantified the spatial intraspecific alteration of functional traits in two bumblebee species. The traits we quantified are relevant for bumblebee biology because they provide information about flight performance and are considered good indicators of stress during bumblebee development (Gérard et al. 2018b; Greenleaf et al. 2007; Klingenberg et al. 2001). We focused on the morphological variations of *B. terrestris* and *B. pascuorum* along an urbanization gradient and our results highlighted some correlations between stressors related to urbanization, and traits as wing size and wing size asymmetry. Specifically, the landscape temperature and the abundance of floral resources, two environmental features influenced by the degree of urbanization (See online resources Table S1, Additional information 1, and Table S2), emerged as candidate drivers of intraspecific variation of size across bumblebee populations, acting differently on the two investigated species. Foragers of *B. pascuorum* showed a shift toward smaller body size in response to increasing temperature, a condition often associated with deeply urbanized landscapes, generally referred to as the heat island effect (Chun and Guldmann 2018). Although a similar pattern of body size reduction in urban bumblebees has previously been reported by Eggenberger et al. (2019), the authors did not directly evaluate the effect of temperature. The possible relationship between bumblebee size and temperature has been investigated in historical series from museum collections and in experimental studies that revealed how higher environmental temperatures represent a driver of body size reduction (e.g., Nooten and Rehan 2020; Theodorou et al. 2021). Higher temperature accelerates larval development in insects, which likely results in smaller adults (Sibly and Atkinson 1994). Furthermore, smaller sizes in warmer areas could also be a strategy for reducing overheating risks while foraging, due to an increased convective heat loss in smaller bees (de Farias-Silva and Freitas 2020). Functionally, smaller bumblebee foragers could travel shorter foraging distances (Greenleaf et al. 2007) and could also load less pollen and nectar (Goulson et al. 2002). As a consequence, the shift toward smaller body size in *B. pascuorum* could imply that it will pollinate less plants or handle flowers less efficiently (Földesi et al. 2020), a concerning aspect in view of colony provision and pollination. It is not known if microclimatic conditions of lower temperatures could mitigate the effects we observed, as we used temperature measured at a broader scale. Furthermore, it is important to underline that other landscape variables could act synergically with urban temperature. Further investigations considering microclimatic variations (e.g., using data loggers at each sampling site) and field experiments pointing at cause-effect relationship between temperature and body size will be required to exclude the possible role of other urban related stressors (Piano et al. 2020). The role of different types of impervious surface (e.g., concrete, or buildings, or asphalt) in contributing to temperature increase should also be addressed in future research, to further inform mitigation strategies in urban contexts.

Size reduction was also previously explained by the decrease in floral resource abundance in urban landscapes, possibly as a consequence of reduction of green areas (Merckx et al. 2018; Eggenberger et al. 2019). According to this evidence, we found a correlation between *B. terrestris* size and the abundance of flowers, with larger individuals observed where more resources were locally available. This is in accordance with the observation that bumblebee adult size is strictly correlated with the amount of food received during larval development (Couvillon and Dornhaus 2009). However, this trend seems to be not clearly confirmed by *B. pascuorum* probably due to a possibly higher flower specialization of this species (Harder 1985). In other words, the narrower diet of *B. pascuorum* could prevent this species from utilizing all the available flowers. Considering the correlations we obtained, future research avenues assessing the role of local resources on bumblebee biology will be important. For instance, investigating other important features such as the nutritional quality of available resources, their diversity, and changes along landscape variation will clarify aspects of nutritional ecology (Vaudo et al. 2015, 2016). Moreover, a more comprehensive characterization of plant communities in space and time will likely provide additional evidence on how flower resources may impact pollinator traits.

Despite *B. pascuorum* and *B. terrestris* belonging to the same genus, they showed a different susceptibility toward the investigated stressors. This suggests that different responses are likely to come from different behavioral features. Idiosyncratic responses were also observed in other bumblebee species, where body size decreased over warming decades in some cases, but others responded in the opposite way (Gérard et al. 2020). In our study the invariant size of *B. terrestris* in warmer conditions could be explained by its higher heat tolerance (Martinet et al. 2020). In addition, *B. terrestris* nests further underground compared to *B. pascuorum*, and it might be less exposed to warm air temperatures during larval development. These aspects strengthen the hypothesis that temperature could be a major determinant of pollinator size reduction in cities because they affected the body size of the more temperature-sensitive species, but not the heat-tolerant one. These idiosyncratic species-specific responses are very relevant for understanding the potential mechanism of intraspecific trait variation associated with urbanization and supports the need to consider a wider panel of species in this kind of studies.

Regarding wing asymmetry we found in *B. terrestris* that size FA was positively correlated with increased temperatures. Variation in both wing size and wing shape asymmetry was observed in other insect taxa and the effect of temperature was previously investigated under controlled laboratory conditions (Mpho et al. 2002). Studies associated the increased wing size and shape FA to environmental stressors, indicating that impairment of developmental processes might take place (e.g., Klingenberg et al. 2001; Kerr et al. 2013). The absence of variation in shape asymmetry registered for both the bumblebee species could confirm the results from other studies that have indicated shape variation as less susceptible to stressors than size asymmetry (e.g., Gérard et al. 2018b). Importantly, floral diet could represent a possible mitigation of environmental stressors during bees’ development (Archer et al. 2014). Here, this view is supported by the negative correlation found between resource abundance and wing size FA, although only in *B. pascuorum*.

Flight performance largely depends on body size and it is also affected by asymmetries in shape and size between wings (Grilli et al. 2017; Soule et al. 2020). Variation in these traits does not only show developmental instability, but also has ecological implications for bumblebees. Indeed, body size is determinant in predicting dispersal ability of bumblebees, thus influencing their foraging range (Greenleaf et al. 2007). Similarly, wing size FA impacts the management of lengthy flights (Fernandez et al. 2017; Soule et al. 2020), while wing shape FA is often associated with flight maneuverability. The combination of these morphological changes could deeply impact flight performance, flight range in time and space, and consequently bumblebee foraging (Kenna et al. 2021), with potential consequences on the pollination service efficiency they provide. However, an important aspect to consider is that wing size and asymmetry could even determine behavioral changes. For example, insects could increase visitation rates at closer distances to colonies, and even spend a higher time on the available resources instead of flying at a broader distance (Andrieu et al. 2009). Such changes, albeit difficult to quantify in the field, could merit further investigation when trying to forecast the impact of functional trait changes in response to urbanization.

## Conclusions

This study suggests that the environmental changes associated with urbanization could affect different functional traits of pollinators, and that their impact occurs heterogeneously on different species. Eventually, as the studied traits are often involved in flying abilities, these responses could bring to the alarming outcome of decreased foraging efficiency and pollination effectiveness in bumblebees. Furthermore, the different responses to the same stressor of the two bumblebees underline the necessity to consider wider panels of taxa in future studies. This strategy will permit to better address the life-history differences among pollinators and thus drive more realistic conclusions.

From a conservation perspective, the comprehension of how pollinators cope with the challenging conditions occurring in novel anthropogenic habitats, plays a key role in informing suitable policy efforts to conserve their biodiversity and the ecosystem service they provide. In the future, cities are predicted to expand constantly and thus designing of urban landscapes will become a fundamental step for achieving sustainability outcomes. The pollinator-friendly design and management of urban green spaces will possibly create suitable conditions for pollinators and thus for the ecosystem services they provide (Guenat et al. 2019; Tommasi et al. 2021). At the same time, urban forestry and greenery practices (e.g., plantation of street and residential trees and the creation of urban greenbelts or greenways) could represent a valid solution to mitigate stressful conditions related to the urban environment, such as the lack of floral resources and the heat island effect (Chun and Guldmann 2018) that here were found to influence functional traits.

## Supplementary Information

Below is the link to the electronic supplementary material.

Supplementary file1 (DOCX 240 KB)

## Data Availability

All relevant data are within the paper or stored in a public repository (https://doi.org/10.6084/m9.figshare.13637594).
