# Supplementary material for: Effect of urbanization and its environmental stressors on the intraspecific variation of flight functional traits in two bumblebee species
Source: Oecologia. 2022 May 16;199(2):289–99. doi: 10.1007/s00442-022-05184-x (PMC9225972; doi:10.1007/s00442-022-05184-x)
Supplement: Supplementary file 1 — Supplementary file1 (DOCX 240 KB) [file 442_2022_5184_MOESM1_ESM.docx]

**ONLINE RESOURCES**

**Effect of urbanization and its environmental stressors on the intraspecific variation of flight functional traits in two bumblebee species**

Nicola Tommasi ^1^, Emiliano Pioltelli ^1^, Paolo Biella ^1^, Massimo Labra ^1, 2^, Maurizio Casiraghi ^1^, Andrea Galimberti ^1, 2^*

1 -ZooplantLab, Department of Biotechnology and Biosciences, University of Milano-Bicocca, Milano, Italy

2 -INFN Sezione di Milano Bicocca, Milano, Italy

*Correspondence: [andrea.galimberti@unimib.it](mailto:andrea.galimberti@unimib.it)

**Table of contents**

**Table S1 -** Table of variables distribution among sampling sites **– pag 2**

**Additional information 1 -**Bar plots representing variables distribution among sampling sites **– pag 3-7**

**Table S2 -** Correlation matrix between variables **– pag 7**

**Figure S1 -** Map of mean temperature and NO_2_ sampling points **- pag 8**

**Table S3 -** Complete regression models outputs **– pag 9**

**Additional information 2 -** Description of DUSAF levels categorised as “Impervious” and “Semi Natural” **– pag 10**

**Additional information 3 -** Computing of Procrustes ANOVA degrees of freedom

**TABLE S1 - TABLE OF VARIABLES DISTRIBUTION AMONG SAMPLING SITES**

| **SITE CODE** | **Impervious/semi natural surfaces** | **Edge density** | **Min C°** | **Average C°** | **Max C°** | **Min NO_2_ µg/m3** | **Average NO_2_ µg/m3** | **Max NO_2_ µg/m3** | **Floral resources** |
| --- | --- | --- | --- | --- | --- | --- | --- | --- | --- |
| 1 | 0.0 | 0.040 | 28.6 | 30.3 | 32.7 | 7.1 | 22.44 | 41.100 | 510 |
| 2 | 0.1 | 0.045 | 28.4 | 30.6 | 33.2 | 10.4 | 26.87 | 49.800 | 823 |
| 3 | 0.0 | 0.020 | 27.2 | 29.9 | 32.8 | 2.0 | 15.25 | 32.800 | 198 |
| 4 | 0.1 | 0.055 | 28.6 | 30.5 | 32.2 | 2.0 | 15.25 | 32.800 | 1001 |
| 5 | 0.0 | 0.043 | 27.7 | 29.9 | 31.9 | 2.0 | 15.25 | 32.800 | 141 |
| 6 | 0.0 | 0.069 | 30.9 | 33.1 | 35.5 | 8.0 | 18.17 | 42.000 | 490 |
| 7 | 1.9 | 0.088 | 29.6 | 32.0 | 33.8 | 10.7 | 23.82 | 51.900 | 622 |
| 8 | 1.2 | 0.069 | 31.4 | 33.5 | 35.5 | 10.7 | 23.82 | 51.900 | 4049 |
| 9 | 1.5 | 0.077 | 32.8 | 35.1 | 37.5 | 19.0 | 26.12 | 31.000 | 3020 |
| 10 | 8.6 | 0.093 | 36.2 | 38.3 | 40.7 | 16.1 | 30.77 | 52.400 | 662 |
| 11 | 8.5 | 0.063 | 36.1 | 38.1 | 40.5 | 16.1 | 30.77 | 52.400 | 190 |
| 12 | 11.6 | 0.073 | 34.9 | 37.2 | 39.5 | 16.1 | 30.77 | 52.400 | 583 |
| 13 | 5.3 | 0.038 | 35.2 | 37.5 | 40.1 | 16.1 | 30.77 | 52.400 | 583 |
| 14 | 6.7 | 0.051 | 35.2 | 37.7 | 40.3 | 16.1 | 30.77 | 52.400 | 270 |
| 15 | 8.1 | 0.053 | 35.8 | 37.9 | 40.5 | 16.1 | 30.77 | 52.400 | 244 |
| 16 | 14.1 | 0.065 | 36.3 | 38.0 | 39.9 | 16.1 | 30.77 | 52.400 | 577 |
| 17 | 17.4 | 0.081 | 35.6 | 38.4 | 40.8 | 16.1 | 30.77 | 52.400 | 410 |
| 18 | 2.3 | 0.044 | 35.2 | 37.5 | 39.3 | 16.1 | 30.77 | 52.400 | 1273 |
| 19 | 11.8 | 0.083 | 36.1 | 37.8 | 39.5 | 21.3 | 40.89 | 57.600 | 826 |
| 19 | 0.2 | 0.071 | 31.4 | 33.6 | 34.9 | 10.0 | 19.29 | 38.000 | 420 |
| 20 | 1.5 | 0.046 | 34.9 | 36.5 | 37.9 | 21.6 | 39.94 | 53.500 | 248 |
| 21 | 1.5 | 0.023 | 34.2 | 36.0 | 38.0 | 21.3 | 40.89 | 57.600 | 201 |
| 22 | 2.9 | 0.086 | 35.3 | 37.4 | 39.5 | 31.0 | 38.19 | 55.000 | 1805 |
| 23 | 1.3 | 0.069 | 33.9 | 35.4 | 36.9 | 12.0 | 17.62 | 29.000 | 420 |
| 24 | 1.3 | 0.057 | 31.7 | 33.5 | 35.1 | 12.0 | 17.62 | 29.000 | 664 |
| 25 | 0.4 | 0.096 | 33.1 | 35.3 | 36.8 | 12.0 | 17.62 | 29.000 | 612 |
| 26 | 0.5 | 0.036 | 31.5 | 33.7 | 35.6 | 15.0 | 22.69 | 28.000 | 592 |
| 27 | 1.4 | 0.069 | 33.9 | 37.0 | 39.0 | 14.0 | 17.5 | 25.000 | 4270 |
| 28 | 1.1 | 0.085 | 32.5 | 34.4 | 35.8 | 19.1 | 32.06 | 49.700 | 765 |
| 29 | 0.1 | 0.083 | 31.6 | 33.5 | 34.8 | 19.1 | 32.06 | 49.700 | 261 |
| 31 | 0.2 | 0.063 | 28.6 | 30.5 | 33.0 | 6.0 | 10.15 | 17.000 | 487 |
| 32 | 0.1 | 0.035 | 27.2 | 28.6 | 31.2 | 6.0 | 10.15 | 17.000 | 703 |
| 33 | 1.1 | 0.089 | 28.5 | 31.0 | 33.0 | 6.4 | 12.81 | 22.300 | 504 |
| 34 | 0.2 | 0.053 | 29.2 | 31.5 | 33.3 | 1.5 | 7.84 | 15.400 | 262 |
| 35 | 0.3 | 0.056 | 29.3 | 30.9 | 32.2 | 6.3 | 14.22 | 23.900 | 238 |
| 36 | 0.5 | 0.075 | 28.0 | 30.6 | 32.6 | 6.3 | 14.22 | 23.900 | 774 |
| 37 | 0.7 | 0.061 | 29.9 | 31.9 | 35.2 | 5.0 | 9.24 | 12.000 | 487 |

**ADDITIONAL INFORMATION 1 - BAR PLOTS REPRESENTING VARIABLES DISTRIBUTION AMONG SAMPLING SITES**


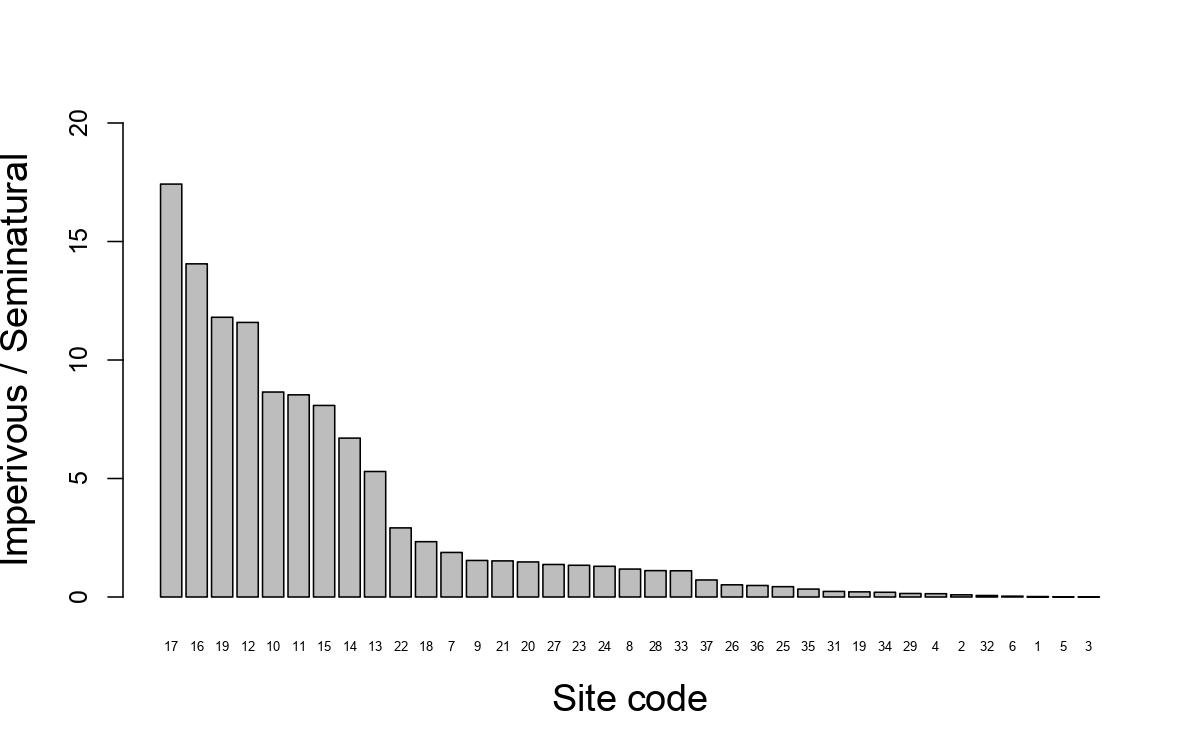


**Additional information 1A:** Impervious/Semi natural ratio along the sampling sites of the urbanization gradient (range 0 -17.4). Data on land cover were retrieved from the regional land use cartography (2018-DUSAF 6.0; <https://www.dati.lombardia.it/Territorio/Dusaf-6-0-Uso-del-suolo-2018/7rae-fng6>).


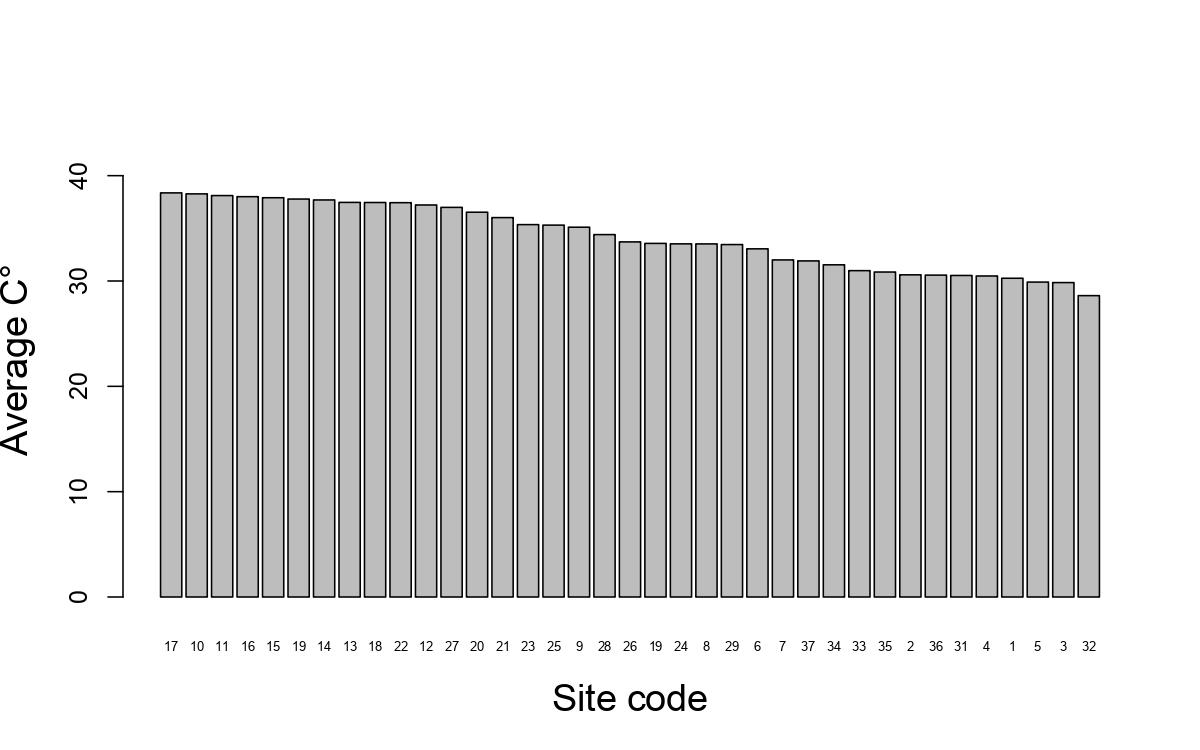


**Additional information 1B:** Distribution of temperature (C°) values along the sampling sites of the urbanization gradient (mean range 28.6° – 38.4°). Data on temperature are retrieved from the NASA database (<https://modis.gsfc.nasa.gov/data/dataprod/mod11.php>)


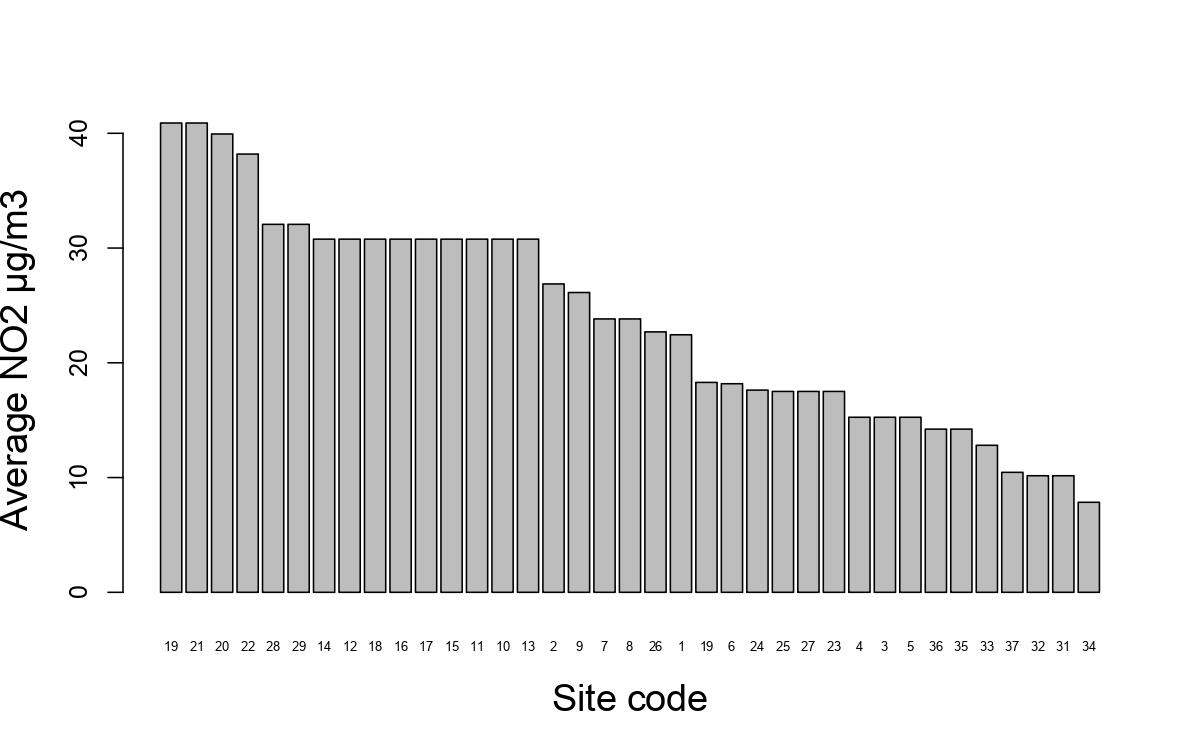


**Additional information 1C:** Distribution of NO_2_ (µg/m^3^) values along the sampling sites constituting the urbanization gradient (mean range 23.7 µg/m3 -40.8 µg/m^3^). Data on NO_2_ produced by the Regional agency environmental protection (ARPA).


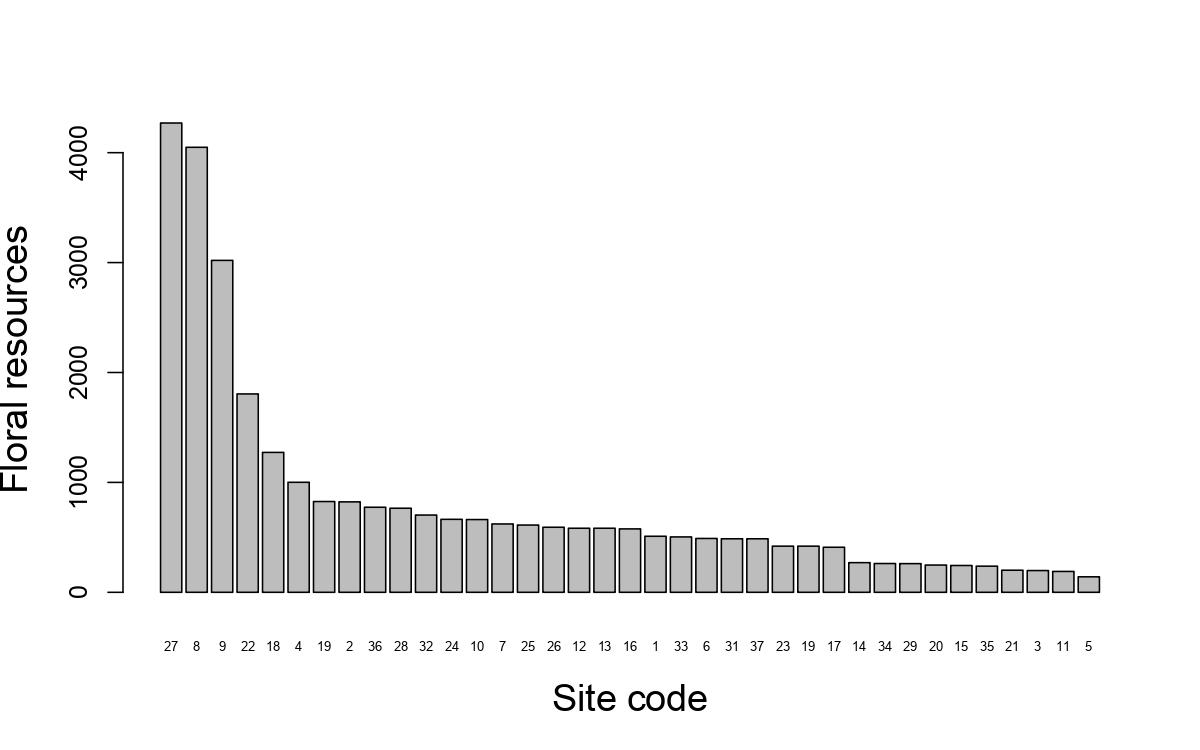


**Additional information 1D:** Distribution of Floral resource availability (estimated number of flowers in standardized units of space) along sampling sites constituting the urbanization gradient (range 141- 4270).


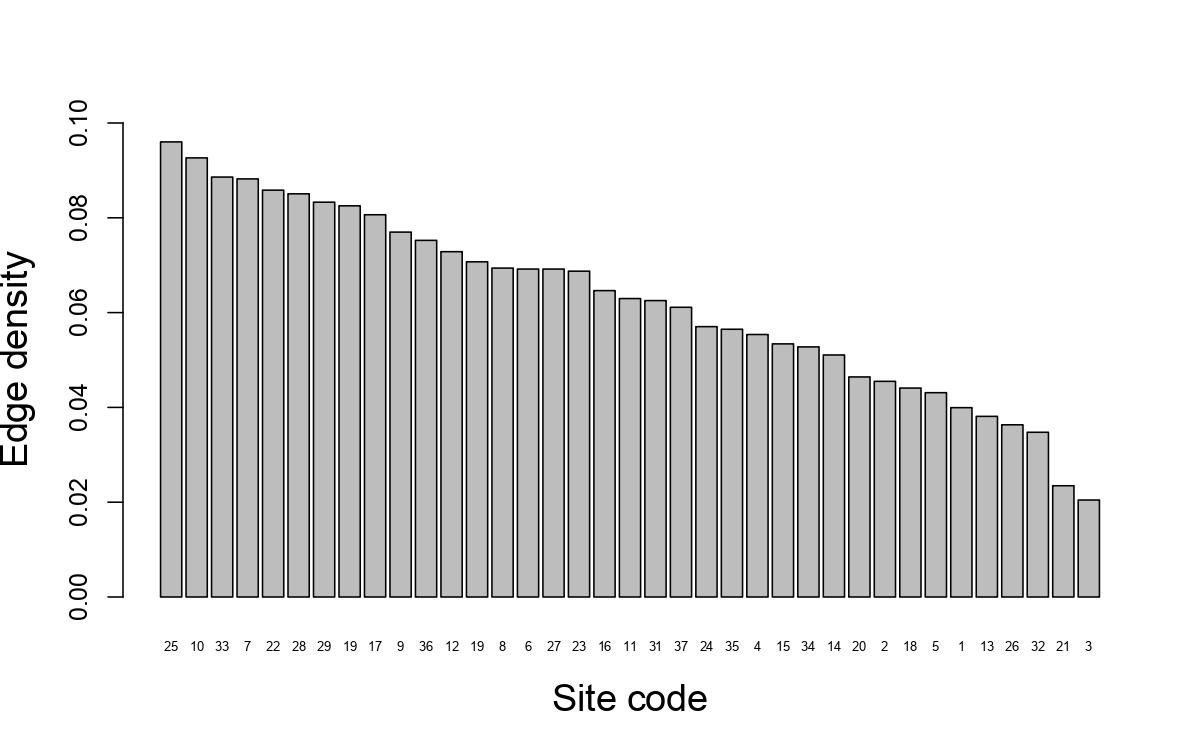


**Additional information 1E:** Distribution of Edge density values (measure of green patches fragmentation) along the sampling sites constituting the urbanization gradient.Data on land cover were retrieved from regional land use cartography (2018-DUSAF 6.0; <https://www.dati.lombardia.it/Territorio/Dusaf-6-0-Uso-del-suolo-2018/7rae-fng6>).

**TABLE S2 - Correlation matrix between variables**

|  | NO_2_ | Edge density | Temperature (C°) | Impervious/natural surfaces | Floral resources |
| --- | --- | --- | --- | --- | --- |
| NO_2_ | **1.00** | **0.09** | **0.75** | **0.64** | **0.02** |
| Edge density | **0.09** | **1.00** | **0.27** | **0.28** | **0.35** |
| Temperature (C°) | **0.75** | **0.27** | **1.00** | **0.84** | **0.12** |
| Impervious/natural surfaces | **0.64** | **0.28** | **0.84** | **1.00** | **0.03** |
| Floral resources | **0.02** | **0.35** | **0.12** | **0.03** | **1.00** |

**FIGURE S1 - Map of mean temperature and NO_2_ sampling points**

Figure S1: The map shows the mean temperature in the may-july period. The location from where NO_2_  values were recovered from the same period are also reported with a triangle. Sampling site locations are pointed by dots.Data on temperature are retrieved from the NASA database <https://modis.gsfc.nasa.gov/data/dataprod/mod11.php>)


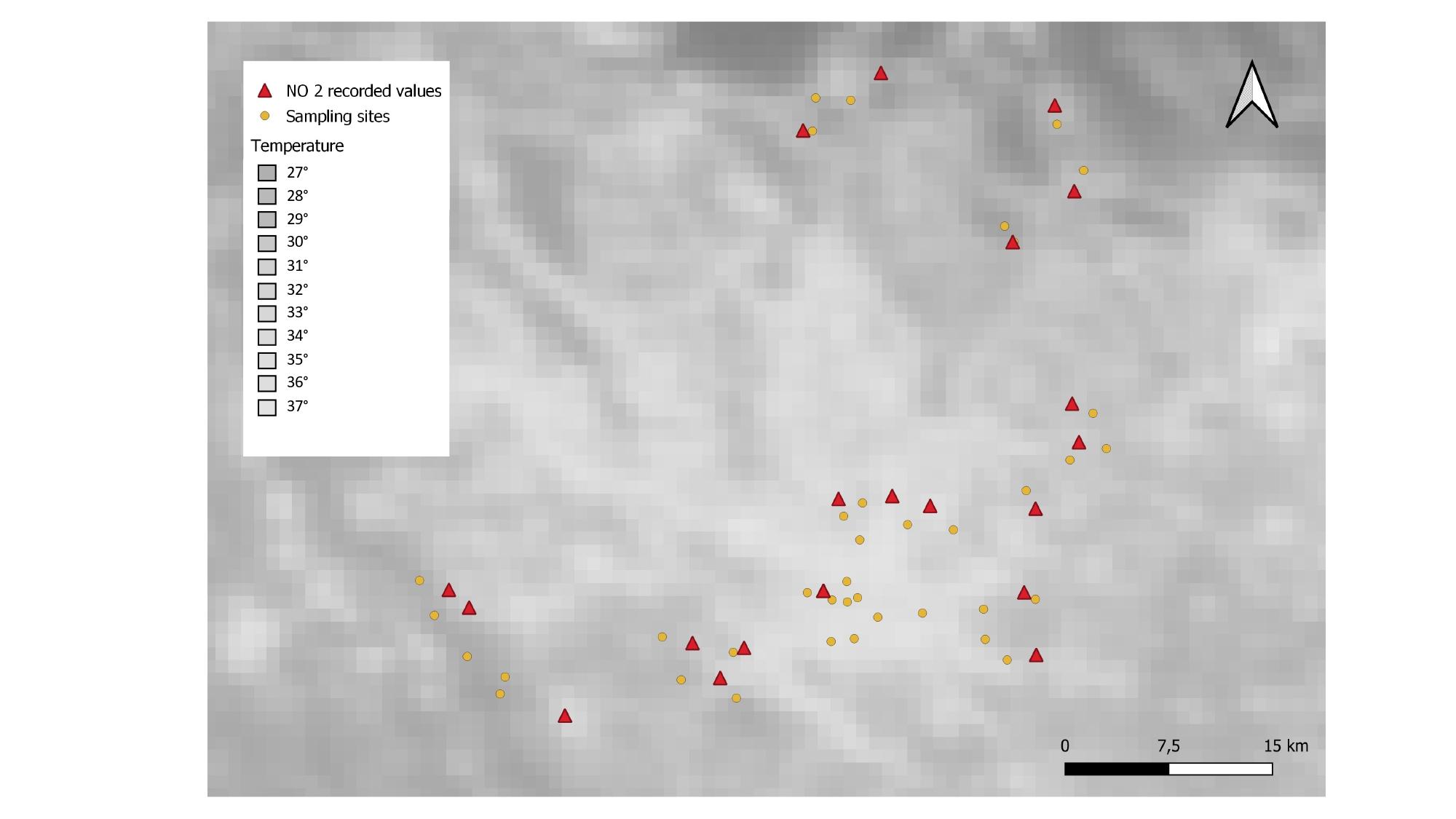


**TABLE S3 COMPLETE REGRESSION MODELS OUTPUTS**

Output of Linear mixed models of body size (N= 348) and fluctuating size asymmetry (size FA) (N= 347) of each species as a function of biotic and abiotic covariates of urbanization, with site identity as random factor. Final models were selected through backward stepwise selection using AIC criterion. ΔAIC reports the difference in AIC values between full and final models. βi: regression coefficient; χ2: chi square values; df: degrees of freedom. Significant p values are reported in bold.

| **Species** | **Response variable** | **Full model covariates** | **Final model covariates** | **ΔAIC** | **B_i_** | **χ2; df** | **p value** |
| --- | --- | --- | --- | --- | --- | --- | --- |
| *Bombus terrestris* | Body size | Temperature | log (Floral resources) | 16.5 | 0.025 | 6.610;1 | **0.010** |
|  |  | Edge density | (1\|Site) |  |  |  |  |
|  |  | log (Floral resources) |  |  |  |  |  |
|  |  | Variables interaction |  |  |  |  |  |
|  |  | (1\|Site) |  |  |  |  |  |
| *B.pascuorum* | Body size | Temperature | Temperature | 17.9 | -0.003 | 7.403;1 | **0.006** |
|  |  | Edge density | (1\|Site) |  |  |  |  |
|  |  | log (Floral resources) |  |  |  |  |  |
|  |  | Variables interaction |  |  |  |  |  |
|  |  | (1\|Site) |  |  |  |  |  |
| *B.terrestris* | Size FA | Temperature | Temperature | 23.5 | 0.052 | 7.183 | **0.007** |
|  |  | log (Floral resources) | (1\|Site) |  |  |  |  |
|  |  | NO_2_ |  |  |  |  |  |
|  |  | Variables interaction |  |  |  |  |  |
|  |  | (1\|Site) |  |  |  |  |  |
| *B.pascuorum* | Size FA | Temperature | log (Floral resources) | 30.3 | -0.161 | 6.118;1 | **0.013** |
|  |  | log (Floral resources) | (1\|Site) |  |  |  |  |
|  |  | NO_2_ |  |  |  |  |  |
|  |  | Variables interaction |  |  |  |  |  |
|  |  | (1\|Site) |  |  |  |  |  |
| *B.terrestris* | Shape FA | Temperature |  |  |  | 0.819;1 | 0.366 |
|  |  | log (Floral resources) |  |  |  | 1.088;1 | 0.297 |
|  |  | NO_2_ |  |  |  | 0.774;1 | 0.379 |
|  |  | Variables interaction |  |  |  | 0.689;1 | 0.407 |
|  |  | (1\|Site) |  |  |  |  |  |
| *B.pascuorum* | Shape FA | Temperature |  |  |  | 2.508;1 | 0.113 |
|  |  | log (Floral resources) |  |  |  | 0.023;1 | 0.881 |
|  |  | NO_2_ |  |  |  | 0.624;1 | 0.429 |
|  |  | Variables interaction |  |  |  | 0.675;1 | 0.411 |
|  |  | (1\|Site) |  |  |  |  |  |

**ADDITIONAL INFORMATION - 2 Description of DUSAF levels categorised as “Impervious” and “Semi Natural”**

List of Level 3 and 4 DUSAF codes Categorized as “Impervious”:

“111”, “1121”, “1122”, “1123”, “1211”, “1212”, “122”, “124”, “131”, “132”, “133”, “134”, “1421”, “1422”, “1423”

and “Seminatural”: “141”, “224”, “231”, “311”, “314”, “322”, “324”, “331”, “332”, “411”.

Full explanation of codes is available at:

<https://www.cartografia.regione.lombardia.it/metadata/Dusaf/doc/Legenda_DUSAF_2018_6_0.pdf>

**ADDITIONAL INFORMATION - 3 Computing of Procrustes ANOVA degrees of freedom**

In order to calculate the degrees of freedom in a Procrustes ANOVA the squared deviations are summed over all the landmark coordinates. In detail, for shape asymmetry the number of degrees of freedom is the number of degrees of freedom of an ordinary ANOVA multiplied by the shape dimension. The shape dimension for two-dimensional data is calculated as twice the total number of landmarks minus four. For example, if we have 40 specimens digitized twice (as in our estimation of the measurement error) the degrees of freedom for the size asymmetry are equal to (40 x 2) -1=79. Conversely for the shape asymmetry it is necessary to take into account the shape dimension; the degrees of freedom are then equal to 79 x (15 x 2 - 4) = 2054. For detailed information on the Procrustes ANOVA see Klingenberg (2015).

Klingenber, C. P. (2015). Analyzing Fluctuating Asymmetry with Geometric Morphometrics: Concepts, Methods, and Applications. *Symmetry*, 7(2), 843-934. https://doi.org/10.3390/sym7020843
